# Temporal trends in epidemiology and patient characteristics of 36 cancers: a protocol for a multinational population-based cohort study using OMOP-standardised databases to investigate CANcer (OMOPCAN)

**DOI:** 10.1136/bmjopen-2026-119069

**Published:** 2026-07-21

**Authors:** Irene López-Sánchez, Anna Palomar-Cros, Agustina Giuliodori, Laura Granés, Laura Pérez-Crespo, Berta Raventós, Edward Burn, Anton Barchuk, Annelies Verbiest, Caroline Eteve-Pitsaer, Danielle Newby, Elin J Rowlands, Espen Enerly, Gargi Jadhav, Harlinde De Schutter, Jason C Hsu, Jelle Evers, Josip Vrancic, Laia Peruchet- Noray, Lillian Sung, Loretta Kiss, Maaike Van Swieten, Mario Šekerija, Mees Mosseveld, Patricia L Mabry, Raivo Kolde, Samuel Patnoe, Seng Chan You, Subin Kim, Sulev Reisberg, Thi Kim Hien Nguyen, Toni Lehtonen, Tor Åge Myklebust, Zsolt Bagyura, Asieh Golozar, Elena Roel, Talita Duarte-Salles

**Affiliations:** 1 Fundació Institut Universitari per a la recerca a l’Atenció Primària de Salut Jordi Gol i Gurina (IDIAPJGol), Barcelona, Spain; 2 Programa de Doctorat en Metodologia de la Recerca Biomèdica i Salut Pública, Universitat Autonoma de Barcelona, Barcelona, Spain; 3 Department of Medical Informatics, Erasmus University Medical Center Rotterdam, Rotterdam, Netherlands; 4 Health Data Sciences (HDS), Translational Sciences, Botnar Research Centre, University of Oxford, Oxford, UK; 5 Department of Medical Oncology, Antwerp University Hospital, Edegem, Belgium; 6 MIPRO, University of Antwerp, Antwerp, Belgium; 7 Cegedim Health Data, Boulogne-Billancourt, France; 8 Department of Registration, Norweigan Institute of Public Health, Cancer Registry of Norway, Oslo, Norway; 9 IQVIA Real World Solutions, London, UK; 10 Belgian Cancer Registry, Brussels, Belgium; 11 International Ph.D. Program in Biotech and Healthcare Management, College of Management, Taipei Medical University, Taipei, Taiwan; 12 Clinical Data Center, Office of Data Science, Taipei Medical University, Taipei, Taipei City, Taiwan; 13 Department of Research and Development, Netherlands Comprehensive Cancer Organisation (IKNL), Utrecht, Netherlands; 14 Department of Haematology, Oncology and Clinical Immunology, General Hospital Varazdin, Varazdin, Croatia; 15 Department of Epidemiology and Biostatistics, School of Public Health, Imperial College London, London, England, UK; 16 Division of Haematology/Oncology, The Hospital for Sick Children, Toronto, Ontario, Canada; 17 Institute for Clinical Data Management, Semmelweis University, Budapest, Hungary; 18 Department for Malignant Diseases with Registry, Croatian Institute of Public Health, Zagreb, Croatia; 19 Andrija Stampar School of Public Health, School of Medicine, University of Zagreb, Zagreb, Croatia; 20 HealthPartners Institute, Bloomington, Minnesota, USA; 21 University of Tartu, Tartu, Tartu County, Estonia; 22 Department of Biomedical Systems Informatics, Yonsei University College of Medicine, Seoul, Korea (the Republic of); 23 Yonsei Institute for Innovation in Digital Health, Yonsei University, Seoul, Korea (the Republic of); 24 STACC, Tartu, Estonia; 25 PhD Program in School of Nutrition and Health Sciences, College of Nutrition, Taipei Medical University, Taipei, Taiwan; 26 Data and Analytics, Finnish Institute for Health and Welfare (THL), Helsinki, Finland; 27 Department of Research and Innovation, Møre and Romsdal Hospital Trust, Ålesund, Norway; 28 Nemesis Health, New York, New York, USA

**Keywords:** Cancer, Observational Study, Epidemiology

## Abstract

**Abstract:**

**Introduction:**

Cancer registries remain the gold standard for global cancer monitoring, yet complementing them with electronic health records and claims can significantly enhance the understanding of the cancer burden by providing a more complete picture of the patient journey. The main aim of this project is to serve as a proof of concept for using real-world data mapped to the Observational Medical Outcomes Partnership (OMOP) common data model (CDM) to monitor cancer epidemiology over time and characterise patients’ clinical history and outcomes.

**Methods and analysis:**

This study will be conducted as an observational cohort study using a multinational network of large real-world data sources mapped to the OMOP CDM. Electronic health records (EHR) from primary and secondary care, health insurance claims and cancer registry data will be included. To date, 20 databases from 16 countries, mainly from Europe but also North America and Asia, have committed to participate in the project.

We will investigate the temporal trends in incidence, prevalence and survival of 36 cancers across haematopoietic and solid tumours from 2000 (or the start of accurate data if later) to the last year with complete data. Data from all individuals registered in each of the participating data sources will be eligible for inclusion in the study. For primary care EHR and claims, individuals will be required to have at least 1 year of prior observation to ensure the identification of incident cases and adequate capture of patient characteristics. We will estimate crude and age-standardised incidence and 5-year partial prevalence. Additionally, we will estimate crude and age-standardised overall survival at 1, 5 and 10 years for the total study period and by diagnosis year groups defined according to data availability. All study objectives will be investigated at the database level, with results stratified by age and sex. For incidence and survival analyses, additional stratifications will be performed by clinical conditions and smoking status (where available). We will use the National Cancer Institute (NCI) Joinpoint Regression Programme to model overall trends in cancer incidence and the NCI JPSurv software to estimate trends in survival. Finally, we will characterise individuals diagnosed with an incident cancer based on demographics, clinical conditions and medication use at different time windows.

Findings will be presented separately for each database and further summarised through descriptive aggregation by country and data source type.

**Ethics and dissemination:**

Each data partner will obtain study approval from their local institutional review boards prior to study execution. Distributed queries will be employed, whereby standardised analytical code is shared and run at each site locally. Deidentified, aggregated results will be returned from all participating sites. A minimum cell count of five will be used when reporting results, depending on each collaborator’s data governance requirements.

All study code will be publicly available, and findings will be submitted to open science journals to promote transparency and reproducibility.

**STRENGTHS AND LIMITATIONS OF THIS STUDY**

- Large sample from broadly representative and heterogeneous settings across multiple geographical regions.
- Longitudinal design including data extending to the most recent years available and covering 36 solid and haematologic cancers.
- Use of data mapped to Observational Medical Outcomes Partnership common data model facilitates the harmonisation of diverse data sources, leveraging their complementary strengths and supporting federated and standardised analysis without sharing individual-level data, ensuring patient privacy.
- As electronic health record and medical insurance claims data are collected primarily for clinical or administrative purposes rather than research, there will be a potential risk of outcome misclassification in the study.
- There will be a potential absence or incompleteness of stratification factors, such as smoking status and clinical conditions, due to a lack of consistent recording across some participating databases.

## Introduction

 Cancer is one of the major causes of mortality worldwide after cardiovascular disease.^1^ The latest GLOBOCAN report estimated that in 2022 there were 20 million new cases of cancer and near 10 million deaths from cancer.^2^ The burden of cancer incidence and mortality is growing worldwide, reflecting both a growing and ageing population as well as increased prevalence of the main risk factors.^3^ The continuous surveillance and monitoring of trends in cancer risk factors, incidence, prevalence and long-term outcomes, including survival, are essential for the development, implementation and evaluation of health policies aiming to reduce the burden of disease.

Several initiatives and institutions provide up-to-date global cancer statistics and promote high-quality cancer data collection, including the European Cancer Information System, the CONCORD studies, the GLOBOCAN and NORDCAN projects of the Global Cancer Observatory at the International Agency for Research on Cancer (IARC), the International Association of Cancer Registries and the European Network of Cancer Registries, and more recently, the EU Joint Action CancerWatch.^4–10^

Cancer registries serve as the foundation for most global cancer monitoring as they provide high-quality, systematically collected data on cancer diagnoses. However, they often lack comprehensive information on the full oncology patient journey, including risk factors, comorbidities, prior medications and long-term outcomes beyond survival.^11^ Conversely, electronic health records (EHRs) and health insurance claims cover a wider clinical spectrum, capturing longitudinal medical history, including diagnosis and medication use and enabling a more comprehensive view of the patient journey.^12^ However, they are often less standardised and specifically for primary care and claims, they are less clinically detailed than cancer registries, often lacking information on tumour stage, histology, biomarkers or cancer-specific treatment details.^13–15^ Analysing real-world data from cancer registries together with EHRs and claims could enrich our understanding of the global cancer burden by leveraging the complementary strengths of each data source.

Existing surveillance systems, like the initiatives mentioned above, rarely achieve this level of harmonisation of disparate data sources due to substantial technical barriers, as heterogeneity in data architectures and source vocabularies complicates the development of computationally efficient, reproducible and reliable analytical pipelines.^16^ To mitigate these challenges, the use of a Common Data Model (CDM) offers a robust framework for harmonising heterogeneous data sources across diverse healthcare settings. Specifically, the Observational Medical Outcomes Partnership (OMOP) CDM was designed to standardise the structure, content and semantics of observational data, enabling standardised analytical code to be developed once and executed locally at each data site.^17^ The inception of OMOP promoted the formation of the Observational Health Data Sciences and Informatics (OHDSI; www.ohdsi.org) community. This multi-stakeholder and interdisciplinary international network is composed of more than 4000 collaborators from 83 countries and generates open science through large-scale analytics of real-world data mapped to the OMOP CDM.^18^

In this research context, the OHDSI data network offers a unique opportunity to study cancer epidemiology—cancer incidence, prevalence and survival—while capturing the complexities of patient medical history, such as baseline comorbidities and medication use. A global network study using the OMOP CDM could serve as a valuable proof-of-concept for how distributed network analyses can complement existing surveillance systems, such as the aforementioned global initiatives. By enabling federated analyses of diverse real-world data sources, this approach incorporates informative data to better capture the complete patient journey.

### Objectives of the study

The main aim of this project is to serve as a proof-of-concept for using diverse real-world data mapped to the OMOP CDM to monitor cancer epidemiology and characterise patient clinical history and outcomes. We will evaluate the feasibility and utility of this distributed network approach by estimating temporal trends for incidence, prevalence and survival across 36 distinct cancers and examining the longitudinal medical history of these patients.

The specific study objectives are as follows:

1. To estimate crude and age-standardised incidence rates and 5-year partial prevalence of cancer.
2. To estimate crude and age-standardised overall, 1-year, 5-year and 10-year overall survival rates of cancer.
3. To estimate temporal trends in cancer incidence and survival.
4. To describe the demographic and clinical characteristics, as well as medication use, of individuals at the time of diagnosis and during the periods preceding and following it.

All study objectives will be investigated at the database level, with results stratified by age and sex. For incidence and survival analyses, additional stratifications will be performed by clinical conditions and smoking status (where available). Findings will be presented for each database and further summarised through descriptive aggregation by country and data source type.

## Methods and analysis

### Study design and data sources

This study will be conducted as a multinational observational cohort study through a network of real-world data sources mapped to the OMOP CDM. The recruitment of data sites is being conducted through direct requests to data holders and announcements within the OHDSI community through the oncology working group and the annual Global, European and Asian OHDSI symposiums. As of June 2026, the study network comprises 20 databases from 16 countries, including EHR (primary and secondary care), health insurance claims and cancer registry data (table 1). These databases are located in Belgium, Canada, Croatia, Estonia, Finland, Hungary, Italy, the Netherlands, Norway, Romania, South Korea, Spain, Switzerland, Taiwan, the UK and the USA. The study is planned to run from May to September 2026, including the statistical analysis and the start of dissemination of the results.

**Table 1:** Databases committed to participate as of June 2026

Database name	Country	Type of data	Healthcare setting	Observation start in data source*	Persons in data source*	Mortality, source of mortality
Belgian Cancer Registry—Use case breast and lung cancer (BCR – BALC)	Belgium	Registry	Population-based Cancer Registry	2014	210.9K	Yes, from the Crossroads Bank for Social Security (CBSS) Belgium
Clinical Practice Research Datalink (CPRD GOLD)	UK	EHR	Primary care	1993	17.52 M	Yes, General Practitioners’ Information Systems
Croatian National public health information system (NAJS)	Croatia	Integrated EHR & Registry	Primary care+secondary care (outpatient specialist care and Inpatient care) + Population-based Cancer Registry	2014	4.85 M	Yes, mortality registry
EST-Health-30 (EH30)	Estonia	Integrated EHR & Registry	Population-based Cancer Registry+Secondary Care + Primary Care	2012	488K	Yes, Registry of Causes of Death
Finnish Institute for Health and Welfare (THL)	Finland	Integrated EHR & Registry	Primary care + Secondary care	2011	6.61M	Yes, Finnish Population Information System
Geneva Cancer Registry (GCR)	Switzerland	Registry	Population-based Cancer Registry	1970	123 k	Yes, Federal Statistics Office
HealthPartners	USA	Claims	Secondary Care+Clinics	1994	3.40M	Yes, EHR records and state death data.
Integrated Primary Care Information (IPCI)	The Netherlands	EHR	Primary care	2006	8.55 M	Yes, General Practitioners Information Systems
Netherlands Cancer Registry (NCR)	The Netherlands	Registry	Population-based Cancer Registry	1993	2.63 M	Yes, Personal Records Database
OncEMR	USA	Claims	Secondary Care+Clinics	1939	1.88 M	OncEMR does not capture mortality
Semmelweis University Clinical Data (SUCD)	Hungary	EHR	Secondary Care	1998	2.49M	Planned, Personal Records Database
Taipei Medical University (TMUCRD)	Taiwan	EHR	Secondary Care	2001	3.39 M	Planned, the National Death Registry
The Cancer Registry of Norway (CRN)	Norway	Registry	Population-based Cancer Registry	1953	1.22M	Yes, Norwegian Population Registry
The Health Improvement Network Belgium (THIN BE)-	Belgium	EHR	Primary Care	2008	2.02M	Yes, GP information system connected with insurance
THIN Spain (THIN ES)	Spain	EHR	Primary Care	2010	1.89M	
THIN Italy (THIN - IT)	Italy	EHR	Primary Care	2008	1.22M	
THIN Romania (THIN- RO)	Romania	EHR	Primary Care	2008	1.13M	
The Hospital for Sick Children (SickKids)	Canada	EHR	Paediatric Tertiary Care (Inpatient and Specialist Outpatient)	2018	1.12 M	Yes, hospital-based
The Information System for Research in Primary Care (SIDIAP)	Spain	EHR	Primary Care+Secondary Care Discharge Records	2006	5.95 M	Yes, Regional Registry (Catalan Centre Register of Insured Persons)
Yonsei University Health System (YUHS)	South Korea	EHR	Secondary Care	1993	6.66M	Yes, hospital-based

* Counts represent the total unique individuals present in the data source, including active and inactive individuals.

### Study participants and time at risk

Data from all individuals registered in each of the participating data sources will be eligible for inclusion in the study.

For incidence and prevalence analysis (*objective 1*), the denominator will be derived from the person-time observed within the database in the case of primary care EHR and health insurance claims data. Participants will start contributing person-time to the denominator at the latest of the following: (1) study start date (1 January 2000 or the earliest date of available data in each of the data sources); (2) date at which a 365-day washout period has been completed (to ensure identification of incident cases^19^); (3) date at which they reach a minimum age (when age strata are being considered). Participants will stop contributing person-time at the earliest date of the following: (1) study end date (end of available data in each of the data sources); (2) end of observation period (death or loss to follow-up); (3) date at which they reach a maximum age (when age strata are being considered); or (4) the date of the first record of a cancer diagnosis in the database (for incidence analysis only).

For cancer registries, which only record individuals with the disease and therefore do not capture the underlying population at risk, we will follow IARC standards. This involves approximating the population at risk using mid-year population counts from national statistics to construct denominators.^20^ This approach assumes that persons without the outcome contribute the full year, which we acknowledge it might result in an underestimation of incidence and prevalence. Conversely, for secondary care databases, the underlying population is higher-risk and non-representative of the general population, and the catchment area is poorly defined, preventing the construction of a valid population-at-risk denominator. For this reason, secondary care databases will not contribute to incidence and prevalence analysis, only to survival analyses and patient characterisation.

Participants will contribute to the numerator based on the occurrence of a cancer diagnosis. Incidence estimates will be based on the first occurrence of a cancer diagnosis, whereas the 5-year partial prevalence estimates (also known as limited-duration prevalence) will encompass individuals with a record of the disease observed within the prior 5 years (ie, 1825 days).

For the survival analysis (*objective 2*) only the first recorded cancer diagnosis per cancer type and in each database will be considered. These individuals will be followed from the date of the cancer diagnosis to death, loss to follow-up or end of the study period, whichever comes first. Patients whose death date and cancer diagnosis date coincide, or with multiple primary cancer diagnoses on the same date (unknown primary site), will be excluded from the survival analysis.

### Sample size calculation

Since this study will be undertaken using observational real-world data, we will include all patients meeting the eligibility criteria described above. No prior sample calculation will be performed. The approximate total number of persons captured in each participating database is specified in table 1.

### Outcome definitions

We will investigate 36 cancers: solid tumours in the anus, bladder, brain, breast, cervix, colon, corpus uteri, oesophagus, gallbladder, hypopharynx, kidney, larynx, lip and oral cavity, liver, lung, skin (melanoma and non-melanoma), nasopharynx, oropharynx, ovary, pancreas, penis, prostate, rectum, salivary gland, stomach, testis, thyroid, vagina, and vulva, as well as Hodgkin lymphoma, Kaposi sarcoma, leukaemia, mesothelioma, multiple myeloma and non-Hodgkin's lymphoma.

We used the OMOP CDM standard vocabularies for cancer diagnostic codes: the Systematized Nomenclature of Medicine Clinical Terms (SNOMED CT) and the International Classification of Diseases for Oncology Edition 3 (ICDO-3). In our preparatory work for this study, we conducted an exhaustive research process to define the codes used to identify cancer cases. This algorithm involved identifying candidate codes in SNOMED and ICDO-3 that aligned with GLOBOCAN definitions (based on ICD-10 codes) using the R software package CodelistGenerator.^21^

**Figure 1:**
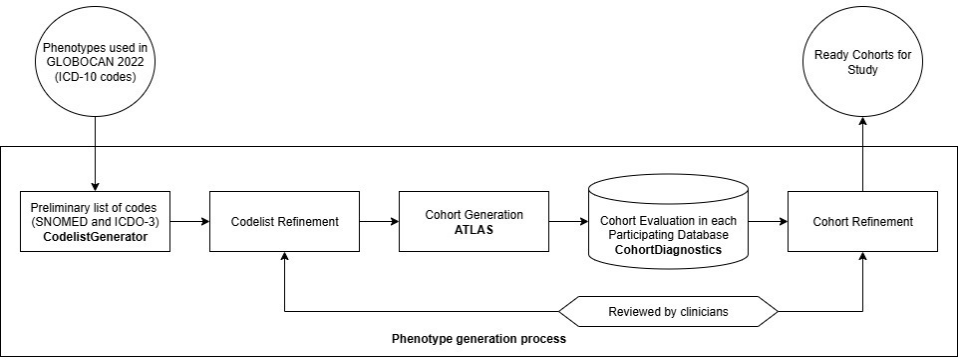
Phenotype generation process. GLOBOCAN, Global Cancer Observatory. ICD-10, International Classification of Diseases 10th Revision. SNOMED, Systematized Nomenclature of Medicine.

We subsequently created cohorts using the open-source OHDSI ATLAS software,^22^ and multiple clinicians (AB, AG, ER and LG) reviewed them (figure 1). We excluded diagnostic codes indicative of in situ, non-invasive, non-malignant, unknown behaviour or metastasis, while neuroendocrine cancers were included. The preliminary list of included codes is provided in online supplemental appendix 1 of the supplementary material. We will further refine this list during the cohort diagnostics phase of the analysis (see more details in the outcome evaluation).

We will estimate overall survival using information on date of death when available (see mortality source data for each database in table 1).

### Outcome evaluation

We will evaluate the robustness of our cancer definitions by using the OHDSI CohortDiagnostic package.^23^ For any cohort and data source mapped to the OMOP CDM, this tool systematically identifies code utilisation and detects potential missed codes. In this study, each data partner will execute the diagnostics and review the output locally to identify phenotyping errors. These results will be further examined by clinicians at the coordinating centre to refine and finalise the cancer phenotypes.

### Covariates

All analyses will be stratified by sex and age groups (0–9; 10–19; 20–29; 30–39; 40–49; 50–59; 60–69; 70–79; 80–89; 90–99; 100 and over). In the event of sparse data or small cell counts, adjacent age strata will be collapsed to ensure statistical stability.

For the analysis of incidence and survival, we will further stratify our results for several covariates according to the cancer as specified in table 2. These covariates were selected based on their availability in EHR databases and their clinical relevance, defined using preliminary codelists provided in online supplemental appendix 2 of the Supplementary Materials. These covariates will include autoimmune conditions, asthma, cardiovascular disease, chronic kidney disease, chronic obstructive pulmonary disease (COPD), depression, HIV, human papillomavirus infection (HPV), hypertension, obesity, type 2 diabetes mellitus and viral hepatitis. When available, results will be further stratified by smoking status. Female organ cancers will also be stratified by menopausal status, which will be defined based on age at time of diagnosis as pre-menopausal (less than 50 years) and post-menopausal (50 years or above).

**Table 2:** Stratifications for each cancer

Groups of cancer*	Stratifications
Respiratory system and intrathoracic organs	Asthma, cardiovascular disease, COPD, hypertension, obesity, smoking
Digestive	Cardiovascular diseases, HPV, hypertension, obesity, smoking, type 2 diabetes, viral hepatitis
Male organs	HPV, obesity, smoking
Female organs	Depression, HPV, menopausal status, smoking, obesity, type 2 diabetes
Lymphoid, haematopoietic	Autoimmune disease, HIV, obesity, smoking
Lip-oral cavity oropharynx	HPV, obesity, smoking
Urinary tract	Chronic kidney disease, hypertension, obesity, smoking
Skin	Obesity, smoking
Other	HIV, obesity, smoking

* Cancer groups are based on the standard IARC GLOBOCAN (Global Cancer Observatory) site definitions

COPD, chronic obstructive pulmonary disease; HIV, human immunodeficiency virus; HPV, human papillomavirus.

Additionally, for objective 4, we will further characterise individuals with an incident cancer. These variables will include both pre-specified clinical conditions and medications (online supplemental appendix 2 and 3 of the Supplementary Materials), as well as those identified through large-scale data exploration.

### Statistical analysis plan

All analysis will be performed at the database level, and the findings will be presented for each database and further summarised through descriptive aggregation by country and data source type.

To address *objective 1* we will estimate crude and age-standardised incidence rates (IR) and 5-year partial prevalence of cancer. We will estimate annual and overall IR by dividing incident cancer cases by person-years at risk. For each specific cancer type, individuals will contribute to the denominator until cancer diagnosis, death, or end of the study period. Following IARC rules on multiple primary cancers, each of the 36 outcomes will be assessed separately, counting only the first recorded diagnosis per cancer type but allowing individuals to contribute time at risk to the incidence estimation of a different, subsequent primary cancer in other cancer groups.^24^

We will estimate annual 5-year partial prevalence (also referred to as limited-duration prevalence), defined as the number of individuals alive on December 31^st^ of each year who were diagnosed with a specific cancer within the previous 1825 days. The denominator will consist of the total number of individuals alive and under observation in the study population on the same date. Only the first recorded malignant tumour per cancer type diagnosed during the 5-year period will be considered, irrespective of subsequent recurrences. We will explore doing a sensitivity analysis where an individual will contribute to more than one specific prevalence period in the same cancer type in the event of recurrence. In such cases, if a subsequent diagnosis occurs within the active 5 year window, it will elongate the outcome duration. Finally, an individual with multiple different cancer types may contribute to more than one estimate.

For age standardisation, we will consider the European Standard Population (ESP) 2013 and the World Standard Population (WSP) 2000–2025. To address *objective 2* on cancer survival, the 1-year, 5-year and 10-year crude and age-standardised survival rates will be calculated as the probability of being alive 1, 5 or 10 years after diagnosis, respectively, per year, overall and stratified by pre-defined subgroups. For age standardisation, the same standard populations used for objective 1 will be applied. Individuals will be grouped by year of first recorded cancer diagnosis into time windows to account for potential changes in treatment practices over time (to be defined depending on data availability). Survival and 95% CI will be calculated using data on time at risk of death from any cause with the Kaplan-Meier method.^25^ Results will be reported as plots of the estimated probability of overall survival. This analysis will be conducted only for databases that collect validated all-cause mortality data, either through internal records or via linkage to external sources, such as national or subnational death registries (table 1).

We will use joinpoint modelling to examine the overall time trends in cancer incidence and survival (*objective 3*).^26 27^ The model involves fitting a series of joined straight lines on a logarithmic scale to the trends in the annual rates. The direction and magnitude of the resulting trends are described by the annual percent change (APC), the linear slope across each line segment between two joinpoints. The average annual percent change (AAPC) summarises the overall trend over time using a weighted average of the APCs within the specified period. The default maximum number of joinpoints will be set according to the available observation period per database. In describing the change, the term increase or the term decrease will be used when the APC or the AAPC is statistically significant (p<0.05); otherwise, the term stable will be used.

Finally, for *objective 4* on characterisation of individuals with incident cancer, we will summarise the demographic characteristics and overall follow-up at time of diagnosis, with counts and percentages for categorical variables and median and IQRs for continuous variables.^28^ The clinical characteristics of our study population will be described using two complementary approaches. First, a targeted analysis will be performed on a pre-specified list of clinically relevant conditions and medications (online supplemental appendix 2 and 3). Second, we will conduct a broad exploratory characterisation using all available longitudinal records of diagnosis and prescriptions. Medical conditions will be assessed in predefined time windows relative to the date of cancer diagnosis or index date (> 366 days, 365–1 day before, and on the index date). Drug exposures will be assessed between 365 days to 1 day before, and at index date with an additional window extending from 1 to 90 days post-index date.

All analyses will be performed using standardised tools developed to interact with an OMOP database, including the IncidencePrevalence^29^ and CohortSurvival^30^ R packages. For joinpoint modelling, the NCI Joinpoint Regression Programme will be used to evaluate trends in incidence, whereas JPSurv software will be used for survival trends. This study will be executed in a federated manner; each data partner will execute the study code against their local database containing patient-level data and will share only aggregated results with the coordinating centre. The code for this study will be made publicly available at https://github.com/ohdsi-studies/. The code will be developed using the 4.4 R version.

### Data quality control

Overall data quality of the OHDSI Network databases was assessed locally by each data partner using the data quality dashboard after data mapping to the OMOP CDM^31^ which occurred prior to study recruitment. To further ensure that the oncological data were mapped correctly, the CohortDiagnostics R package^23^ was used to evaluate clinical phenotypes, verify data density and check unexpected errors across the participating sites. This tool also provides a structured overview of each database, including total population size, available observation time, and the versions of the OMOP CDM and vocabularies in use.

### Strengths and limitations of the study

#### Strengths

The main strength of this study is the inclusion of twenty large-scale population and EHR-based databases across multiple countries, capturing diverse healthcare systems, clinical settings and patient populations. Another key strength of this study is its longitudinal design, leveraging prospectively collected real-world data spanning more than 25 years, with follow-up extending until the latest year of data availability in each database. The use of data mapped to OMOP CDM facilitates the harmonisation of diverse data sources, leveraging their complementary strengths and supporting federated and standardised analysis without sharing individual-level data, ensuring patient privacy.

#### Limitations

This study will have some limitations. As EHR data and medical insurance claims are collected primarily for clinical or administrative purposes, there will be a potential risk of outcome misclassification and lag of cancer case diagnoses (prevalent vs incident) or death data. However, for some of these participating databases, cancer diagnosis has been previously validated.^32^ Additionally, we will use the OHDSI CohortDiagnostics tool to assess the quality and consistency of cancer case ascertainment across participating databases and only include sources that have undergone prior validation for data quality. Another potential limitation of the study is the absence or incompleteness of certain key variables, such as smoking status and conditions across some databases. Although we anticipate that the analysis of these factors will not be feasible across all participating databases, it will be possible in a subset of databases within the OHDSI network where this information is available. While the mapping of participating databases to an international standard such as OMOP CDM facilitates overcoming differences in coding practices, heterogeneity across data sources (different healthcare systems, case load, etc.) will limit direct cross-country comparisons. However, these differences provide valuable context for interpreting and understanding the observed variations. Lastly, although the participating databases cover three continents, databases from the Global South are not included, which may limit the global generalisability of our findings. However, this study serves as a proof of concept for how diverse real-world data sources could support and complement future cancer surveillance initiatives. By using the OMOP CDM, we ensure high reproducibility and scalability, facilitating the integration of additional databases as more real-world data sources adopt this common data model over time.

## Ethics and dissemination

### Ethical approval

All data partners executing the study within their data sources will have received institutional review board (IRB) approval or waiver for participation in accordance with their institutional governance prior to study execution.

As of June 2026, ethics approvals have been obtained for most participating databases.

The study will be performed within the legal framework of the Belgian Cancer Registry (BCR). In accordance with the Coordinated Law of 10 May 2015 (art. 138), BCR has a legal task to collect data on cancer, subject it to quality control, process and analyse it, encrypt and store it, report on it, make it accessible for research and protect it. Within the boundaries of this legal framework, BCR is allowed to act without Ethical Committee approval. Additionally, this retrospective study does not fall under the Belgian Law of 7 May 2004 regarding experiments on human persons (art. 3,§2) and is therefore not subject to Ethical Committee approval under this law either.

The use of Clinical Practice Research Datalink (CPRD) data for this study has been approved via the Research Data Governance (RDG) Process of the UK Medicines and Healthcare Products Regulatory Agency (protocol 24_004724).

The Ethics committee of the Croatian Institute of Public Health (HZJZ) has approved the use of data for this study (No. 117-17-25-03).

The use of Est-Health-30 data for this study has been approved by the Estonian Bioethics and Human Research Council (No. 1.1-12/817).

The use of Finnish Institute of Health and Welfare (THL) data for this study has been approved under THL’s internal regulation THL/6288/0.01.00/2023, permit number THL/653/6.02.00/2026

The Geneva Cancer Registry (GCR) has committed to participating in the project, and the protocol is currently under review by the ethics committees.

In a letter dated December 4, 2024, HealthPartners Institute’s Institutional Review Board (IRB) indicated that, based on its review of the study protocol Time Trends in Prevalence, Incidence and Survival of Cancer in the OHDSI Network, the data contributed from HealthPartners does not meet the definition of Human Subjects Research under 45 CFR Part 46; therefore, IRB review and oversight are not required.

The use of the Integrated Primary Care Information (IPCI) data has been approved by the IPCI Review Board (registration no. 8/2023).

The use of Netherlands Comprehensive Cancer Organisation (NCR) data for this study has been approved (No. 24-00228).

The use of the OncEMR data does not require Institutional Review Board review or protocol registration, as the database is fully de-identified.

The use of Semmelweis University data for this study has been approved by the Regional Institutional Scientific and Research Ethics Committee of Semmelweis University (permit number 59/2026).

The use of Taipei Medical University data for this study has been approved by the TMU-Joint Institutional Review Board (project code: N202603014).

Anonymous statistics are provided in accordance with the Cancer Registry Regulations § 3-1, cf. Health Registry Act § 19 third paragraph. The regulations allow the Cancer Registry of Norway (CRN) to provide data considered anonymous.

The use of THIN data has been approved by the THIN Scientific Review Committee (Protocol number 23 012 R3). Given the data are anonymised, ethics approval is not required for the participant THIN databases.

The use of Hospital for Sick Children data for this study has been approved by the Research Ethics Board (No. 3917).

The use of Sistema d’Informació per al Desenvolupament de la Investigació en Atenció Primària (SIDIAP) data for this study has been approved by the Clinical Research Ethics Committee of IDIAP Jordi Gol (project code: 24/001‑P).

The use of Yonsei University Health System (YUHS) data for this study has been approved by the Research Ethics Committee of Severance Hospital, Yonsei University Medical Center (No. 4-2025-0411).

### Agreement and consent

This study will use de-identified data, and no patient-level information will be shared between institutions. As such, informed consent and individual patient agreement are not required. The confidentiality of patient records will be strictly maintained at all times. Data custodians at each site will retain full control over data access and analysis execution. The study will be conducted within a federated and distributed data network, in which standardised analysis code is sent to each participating site, and only aggregate, summary-level results are returned to the coordinating team. There will be no transfer of identifiable or patient-level data at any point. Furthermore, all study packages will implement minimum cell count thresholds to suppress small cell sizes, ensuring compliance with privacy regulations and minimising any risk of re-identification, set to five, subject to modifications by each collaborator’s data governance requirements.

### Dissemination

Open science is a key principle of this study. It involves making research methods, data analytic code, and results openly accessible to the broader scientific community and the public. This approach helps ensure that our findings are transparent, reproducible, and reliable.

The code for the study and aggregated results will be made publicly available on GitHub and through Shiny Apps, respectively.

We will deliver presentations at scientific venues and prepare scientific publications for international scientific peer-review journals. We will publish the results of this study following the International Committee of Medical Journal Editors (ICMJE) authorship guidelines and will report the results following the appropriate Strengthening the Reporting of Observational Studies in Epidemiology (STROBE) checklist and the ESMO GeneRalizability of rEal-world evidence (GROW) guidelines.

The main findings of this project will be shared with non-specialised audiences through social media channels. We will deliver regular press releases at key project stages, distributed via the extensive media networks of the study partners.

## Supplementary material

10.1136/bmjopen-2026-119069 online supplemental file 1

10.1136/bmjopen-2026-119069 online supplemental file 2

## Data Availability

No data are available.
