# Supplementary material for: Temporal trends in epidemiology and patient characteristics of 36 cancers: a protocol for a multinational population-based cohort study using OMOP-standardised databases to investigate CANcer (OMOPCAN)
Source: BMJ Open. 2026 Jul 21;16(7):e119069. doi: 10.1136/bmjopen-2026-119069 (PMC13410706; doi:10.1136/bmjopen-2026-119069)
Supplement: online supplemental file 2 [file bmjopen-16-7-s002.docx]

### **Annex 1. Preliminary code list of cancer definitions**

Attached as a standalone document

### **Annex 2. Preliminary code list of conditions for patient characterisation and stratification**

| Conditions | Concept id (Included) | Concept id (Excluded) |
| --- | --- | --- |
| Alcoholism | 4338024, 201343, 3654690, 37017151, 433753, 374623, 435243, 45757131, 45757093, 37164777, 4176653, 196463, 4045848, 4104707, 193256, 4340385, 195300, 4340386, 201612, 4081520, 4340493, 4102250, 4340964, 36713086, 4176652, 4204015, 4030070, 4028805, 4218106, 4078688, 4120447, 4152403, 4122928, 375519, 377830, 439277, 4202330, 4146660, 46269816, 46269817, 4159583, 37016176, 37017009, 3654681, 439005, 35624505, 45757494, 37016175, 37164785, 436953, 37016167, 378726, 36714559, 37016266, 37162881, 37164781, 435532, 619608, 36715922, 45757783, 36716878, 46269835, 46269818, 37016174, 40483827, 37016173, 4206341, 4338370, 37017329, 37018356, 37164789, 37164792, 37164791, 37164790, 4047762, 37164783, 4152165, 435534, 440685, 441276, 37164793, 607374, 37016172, 37165554, 37109948, 4109691, 37017563, 37164786, 37018663, 4050977, 4195620 |  |
| Anaemia | 439777 | 434701, 43022052 |
| Anxiety | 441542 |  |
| Asthma | 317009, 4235703, 4279553 |  |
| Atrial fibrillation | 313217 |  |
| Autoimmune conditions | 4137275, 81893, 40484648, 201254, 4063582, 134442, 257628, 254443, 4035611, 4083556, 438688, 45772123, 4262578, 2107559, 2107558, 2107560, 80809, 4145240, 46273478, 81931, 140168, 4102493, 46270482, 432295, 2108721 |  |
| Cardiovascular disease | 437894, 4317150, 4148906, 42535425, 43530727, 43530674, 4329847, 316139, 315295, 441874, 443454, 375557, 372924, 321042, 315295 | 4132309, 314666, 442289, 4048809 |
| Cerebrovascular disease | 381591 |  |
| Chronic kidney disease | 194385, 46271022, 192279, 4263367, 261071, 201313, 4103224, 193253, 195314, 192359, 45768812 | 45769152, 195289, 195737, 43530912, 37116834, 195014, 197930, 197320, 4066005 |
| Chronic liver disease | 4212540 |  |
| Chronic Obstructive Pulmonary Disease | 255573, 258780 |  |
| Coronary arteriosclerosis | 317576 |  |
| Crohn’s disease | 201606, 46269889, 46269999 |  |
| Dementia | 37312036, 37312035, 4041685, 37312031, 37312030, 35608576, 4092747, 4182210, 37311665, 4043378, 45765480, 45765477, 37311890, 37312577, 4059191 | 37116464, 37017549, 4244346, 377788, 372610, 37017247 |
| Depressive disorder | 440383 | 438727, 436665, 40481798, 435520, 4224940 |
| Down syndrome | 4159650 |  |
| Gastroesophageal Reflux Disease | 318800 |  |
| Gastrointestinal bleeding | 192671 |  |
| Heart Failure | 316139 | 315295 |
| Human Immunodeficiency Virus (HIV) | 4276586, 44783356, 439727 | 4013105, 432554 |
| Human Papillomavirus infection | 40480043, 4084948, 4116193, 4080771, 4294441, 4291601, 619210, 760929, 4219870, 4266804, 4269876, 4084816, 44810559, 4304732, 4164483, 4129543, 4175989, 198075, 4080330, 4147672, 4078931, 4291605, 37116426, 4347555, 4177636, 4345817, 4148102, 4345473, 36713662, 37206940, 45757380, 36717114, 45757381, 441788, 4333885, 35610330, 36715556, 35610522, 4270602, 37109025, 42535207, 4084817, 4080770, 36716153, 4291602, 4306683, 4300215, 4081909, 4142828, 4185025, 4182586, 4200132, 3657814, 3657815, 4080331, 760906, 760907, 36685421, 44810378, 4302049, 36715819, 4345474, 4145196, 4028324, 137785, 140641, 40490394, 40491348, 40490302, 40489357, 4291600, 4294439, 4300214, 4294440, 4291603, 4130346, 36716496, 4296065, 3656108, 4111926, 4289145 |  |
| Hyperlipidaemia | 432867 |  |
| Hypertension | 316866, 4322024, 42709887 | 4167493 |
| Hyperthyroidism | 140673 |  |
| Ischemic heart disease | 4185932 |  |
| Myocardial infarction | 4329847 | 314666 |
| Non-Alcoholic Fatty Liver Disease | 4026131, 40484532 |  |
| Obesity (BMI and diagnosis) | 36304833^a^, 3038553^a^, 302749^b^, 3023166^b^, 3013762^b^, 3025315^b^, 4099154^b^, 4081038, 4176962, 433736, 45766204, 4256640, 4060985 |  |
| Osteoarthritis | 80180 |  |
| Osteoporosis | 80502 |  |
| Pancreatitis | 4192640 |  |
| Peripheral vascular disease | 321052 |  |
| Pneumonia | 4050869, 255848 | 45770911, 4001167, 4049965, 36712839, 252552 |
| Psoriasis | 140168 |  |
| Pulmonary embolism | 440417 |  |
| Renal impairment | 4030518 |  |
| Rheumatoid arthritis | 80809 |  |
| Stroke | 42535426, 4048784, 4045735, 4031045, 761110, 372924, 4110189, 443454, 762951, 765515, 43530683, 762933, 762937, 4111714, 4108356, 45772786, 4110190, 762935, 763015, 46273649, 35610084, 46270031, 762934, 43531607, 35610085, 46270381, 4110192, 45767658, 44782773, 46270380, 37110678, 37110679, 381316, 35609033, 4046362, 4131383, 4046237, 4119140, 4043731, 439847, 4141405, 37116473, 4144154, 4111709, 4077086, 4046359, 4319146, 4043732, 4146185, 36717605, 43530727, 4148906, 43530728, 432923, 4108952, 4111708, 4142739, 4046358, 36684840 |  |
| Type 1 Diabetes | 36715571, 45769891, 37016767, 45763585, 4128019, 4225656, 45773688, 45773576, 45771075, 45769902, 45769903, 45769837, 35626765, 45769832, 45757674, 435216, 42538169, 42535539, 377821, 37016353, 42689695, 45769904, 43531565, 4221344, 4223303, 37017429, 765533, 37016348, 45757432, 443592, 201531, 42535540, 45757393, 45771067, 45769876, 4228112, 45757362, 3046418, 4047906, 4102018, 45757073, 439770, 4224254, 4143857, 35626069, 45757535, 37016179, 43530660, 37016180, 4225055, 45769829, 45769830, 37312218, 45768456, 45763583, 45769834, 36713094, 318712, 37018566, 4222687, 4222553, 37017431, 4063042, 43531008, 43531009, 45763584, 45757604, 200687, 45757266, 4227210, 45770986, 45771533, 45773567, 45769833, 765373, 46269764, 4143689, 45769873, 201254, 40484648, 40484649, 4152858, 443412, 4099214, 45766051, 45757507, 45769892, 37312201, 45770902, 37312200, 45757074, 4224709, 765650. |  |
| Type 2 Diabetes | 4321756, 36717156, 43531588, 45769888, 4196141, 37016768, 609103, 609106, 609114, 609117, 602345, 45763582, 40483315, 4221495, 43531578, 43531559, 45769901, 43531566, 43531653, 43531577, 43531562, 37309630, 45769894, 43531616, 45757474, 36684827, 37018912, 443732, 43531597, 443733, 376065, 43531564, 45757280, 45769906, 4177050, 4223463, 43530690, 4222876, 37018728, 45772019, 604741, 37016349, 45770880, 201530, 4215719, 45757392, 45771064, 45757447, 45757446, 45757445, 45757444, 45757363, 45772060, 36714116, 608884, 45769875, 4130162, 45757075, 765375, 45771072, 443734, 4228443, 4140466, 45770830, 35626070, 45769905, 45757435, 609099, 609101, 43531651, 45770881, 609104, 609105, 4222415, 37162626, 45769828, 760989, 761063, 43531563, 45757450, 37312203, 37312202, 45770883, 37016354, 43530656, 609096, 609095, 45769836, 443729, 43530689, 45757278, 4221487, 4223739, 37017432, 3192767, 3191208, 3194332, 4063043, 43530685, 609116, 609119, 45770831, 45757499, 443731, 45770928, 4226121, 45769872, 45769835, 761053, 609109, 609112, 36712670, 46274058, 4142579, 45770832, 45773064, 201826, 45757508, 4230254, 4304377, 40485020, 4193704, 4200875, 4099651, 45769890, 37312205, 36712686, 45757277, 37312204, 36712687, 45757449, 43531608, 4099216, 761062 |  |
| Ulcerative colitis | 81893 |  |
| Urinary Tract Infection | 81902 |  |
| Venous thromboembolism | 762047, 762148, 761444, 35616028, 35615035, 761416, 35615031, 43531681, 35616027, 35615034, 761415, 35615030, 44782746, 44782751, 762008, 760875, 765155, 762017, 762417, 762020, 765546, 762004, 44782742, 44782747, 762015, 765541, 44782748, 44782752, 762009, 760876, 765540, 765922, 762418, 765537, 44782767, 46270071, 762022, 44782743, 762021, 762010, 760877, 762013, 762018, 762419, 762005, 44782745, 44782744, 762026, 765156, 44782421, 764016, 44782766, 4120091, 45768439, 45768888, 762048, 45757410, 762049, 36712892, 44782762, 37109253, 40478951, 4042396, 4046884, 4133004, 4181315, 45773536, 763942, 761980, 443537, 4133975, 40480555, 4322565, 763941, 761928, 4207899, 4028057, 435565, 40481089, 4309039, 4119760, 762808, 40480461, 4124856, 4096099, 440738, 4281689, 4284538, 4309333, 4108681, 46285905, 440417, 37109911, 37016922, 43530605, 254662, 4253796, 4121618, 4119610, 46271900, 4236271, 36713113, 35615055, 4033521, 4119607, 4055089, 4327889, 320741, 439838, 4230403, 4069561, 761831, 761830, 761808, 761832, 761809, 4221821, 440750, 4176614, 761821, 761819, 444097, 761820, 761818, 4110339, 4111868, 4110343, 439314, 4109877, 4112171, 4112172, 4250765, 42538533, 44811347, 765049, 4317289, 4203836, 4175649, 4149782, 4153353, 46285904, 444247, 77310, 4189004 |  |
| Viral Hepatitis | 4291005 |  |
| Descendants from all concepts ids will be considered. ^a^Inclusion criteria applied to these concept ids: a BMI measurement between 30 and 60. ^b^Inclusion criteria applied to these concept ids: a body weight measurement and observation between 120 and 200 kg. | | |

####

### **Annex 3. Preliminary code list of medications for patient characterisation**

| **Medications** | **Concept id (Included)** | **Concept id (Excluded)** |
| --- | --- | --- |
| Agents acting on the renin-angiotensin system | 21601782 |  |
| Antibacterials for systemic use | 21602796 |  |
| Antidepressants | 21604686 |  |
| Antiemetics | 21600490 |  |
| Antiepileptics | 21604389 |  |
| Anti-inflammatory and Antirheumatic products | 21603933, 21602722 |  |
| Antineoplastics | 21601387 |  |
| Antipsoriatics | 21602028 |  |
| Antithrombotic agents | 21600961 |  |
| Antivirals for hepatitis C virus | 1501761 |  |
| Antivirals for HIV | 21603180 |  |
| Beta blocking agents | 21601664 |  |
| Calcium channel blockers | 21601744 |  |
| Chemotherapy | 19057483, 35198076, 1314865, 43009039, 19015523, 1329241, 1333357, 40222431, 1337620, 35884377, 1350066, 1390051, 19054825, 19054821, 1310317, 1311078, 1311409, 1311443, 1311799, 19024728, 1315942, 1338512, 1344354, 19104221, 1350504, 1355509, 1395557, 955632, 36879099, 1314924, 19078097, 19078187, 1367268, 19025348, 1391846, 1394337, 1301267, 740067, 1436650, 1305058, 19125635, 1389036, 1309188, 19002912, 35197924, 1378382, 1304919, 19046625, 19093366, 36879010, 19009165, 40166461, 19135793, 19038536, 35197850, 35884368, 19136210, 19056756, 1341149, 19136750, 1437379, 19137385, 1378509, 35603017, 19042545, 40799155, 905078, 19101677, 19006880, 19012543, 19008264, 1308290, 19008336, 36878852, 1343346, 40799197 |  |
| Diuretics | 21601461 |  |
| Drugs for acid related disorders | 21600046 |  |
| Drugs used in addictive disorders | 21604816 |  |
| Drugs for obstructive airway diseases | 21603248 |  |
| Drugs for diabetes | 21600713, 21600744 |  |
| Hormonal Contraceptives | 21602472 |  |
| Immunosuppressants | 21603891 |  |
| Immunotherapy | 1309770, 1536935, 42629079, 1593273, 792844, 19023835, 19013730, 19086176, 37002419, 45892531, 40241969, 37002369, 35200783, 779144, 1536789, 1594034, 19132294, 35604032, 37498261, 1201211, 42799711, 1201501, 42903942, 36026868, 1379969, 1380068, 40238188, 739856, 1536834, 741947, 35200202, 1301307, 746328, 739531, 45892628, 44507676, 40167582, 1718346, 45775965, 1396729, 1302024, 1314273, 1145484, 40224095, 37002368, 746340, 1734429, 19071159, 741578, 792737, 701957, 747052, 741851, 1735539 |  |
| Iron preparations | 21601078 | 21601119 |
| Lipid modifying agents | 21601853 |  |
| Opioids | 21604254 |  |
| Psycholeptics | 21604489 |  |
| Psychostimulants | 21604752 |  |
| Descendants from all concepts ids will be considered. | | |
