# Supplementary material for: Temporal trends in epidemiology and patient characteristics of 36 cancers: a protocol for a multinational population-based cohort study using OMOP-standardised databases to investigate CANcer (OMOPCAN)
Source: BMJ Open. 2026 Jul 21;16(7):e119069. doi: 10.1136/bmjopen-2026-119069 (PMC13410706; doi:10.1136/bmjopen-2026-119069)
Supplement: Supplementary file 3 [file bmjopen-2026-119069.reviewer_comments.pdf]

PEER REVIEW HISTORY

BMJ Open publishes all reviews undertaken for accepted manuscripts. Reviewers are asked to complete a checklist review form and are provided with free text boxes to elaborate on their assessment. These free text comments are reproduced below.

ARTICLE DETAILS

Title (Provisional)

Temporal trends in epidemiology and patient characteristics of 36 cancers: a protocol for a multinational population-based cohort study using OMOP-standardised databases to investigate CANcer (OMOPCAN)

Authors

López-Sánchez, Irene; Palomar-Cros, Anna; Giuliodori, Agustina; Granés, Laura; Crespo, Laura Perez; Raventós, Berta; Burn, Edward; Barchuk, Anton; Verbiest, Annelies; Eteve-Pitsaer, Caroline; Newby, Danielle; Rowlands, Elin J; Enerly, Espen; Jadhav, Gargi; De Schutter , Harlinde; Hsu, Jason C.; Evers, Jelle; Vrancic, Josip; Peruchet- Noray, Laia; Sung, Lillian; Zsuzsa Kiss, Loretta; Van Swieten, Maaike; Šekerija, Mario; Mosseveld, Mees; Mabry, Patricia L; Kolde, Raivo; Patnoe, Samuel; You, Sen Chang; Kim, Subin; Reisberg, Sulev; Nguyen, Kim Hien Thi; Lehtonen, Toni; Myklebust, Tor Åge; Bagyura, Zsolt; Golozar, Asieh; Roel, Elena; Duarte-Salles, Talita

---

VERSION 1 - REVIEW

**Reviewer**	**1**
**Name**	**Mody, Gita N**
**Affiliation**	**The University of North Carolina at Chapel Hill**
**Date**	**09-Apr-2026**
**COI**	**None**

This manuscript presents a planned protocol for a large, multinational, observational cohort study designed to evaluate temporal trends in epidemiology, patient characteristics, and outcomes across 36 cancer types using standardized real-world data sources. The study leverages the Observational Health Data Sciences and Informatics network, incorporating electronic health records (EHRs), insurance claims, and cancer registry data from 20 databases across 16 countries.

The study addresses a highly relevant and timely problem in oncology and population health. While traditional cancer registries have long been the backbone of cancer surveillance, they are often limited to clinical variables (e.g., incidence, stage, histology,

survival) and as the authors note, lack granular data on comorbidities, longitudinal care pathways, and patient-level characteristics. As cancer burden continues to rise globally, there is increasing need for approaches that integrate real-world clinical data to better understand variation.

By proposing this protocol for integration of the data sets, the authors aim to advance how cancer epidemiology is studied across health systems. If successfully implemented, this work has the potential to change health services research and possibly practice, eventually.

VERSION 1 - AUTHOR RESPONSE

Reviewer Comments:

1. This manuscript presents a planned protocol for a large, multinational, observational cohort study designed to evaluate temporal trends in epidemiology, patient characteristics, and outcomes across 36 cancer types using standardised real-world data sources. The study leverages the Observational Health Data Sciences and Informatics network, incorporating electronic health records (EHRs), insurance claims, and cancer registry data from 20 databases across 16 countries.

The study addresses a highly relevant and timely problem in oncology and population health. While traditional cancer registries have long been the backbone of cancer surveillance, they are often limited to clinical variables (e.g., incidence, stage, histology, survival) and, as the authors note, lack granular data on comorbidities, longitudinal care pathways, and patient-level characteristics. As cancer burden continues to rise globally, there is an increasing need for approaches that integrate real-world clinical data to better understand variation.

By proposing this protocol for the integration of the data sets, the authors aim to advance how cancer epidemiology is studied across health systems. If successfully implemented, this work has the potential to change health services research and possibly practice, eventually.

> **Response:** We would like to thank Dr. Gita N. Mody for her positive assessment of our protocol and for recognising the potential impact of the OMOPCAN study on global cancer monitoring.

Additional changes were made to the manuscript in order to comply with BMJ Open submission and formatting requirements. Figures were removed from the manuscript, and figure legends were listed after the reference section. Figure 1 was removed because we did not have the relevant permissions and it was not sufficiently relevant to retain. Table 1 was shortened to two pages by removing the database description column. Finally, we removed the indirect funders that supported the researchers and institutions participating in the study, as the submission system could not capture the additional explanation and the journal requires that the manuscript and submission system remain identical.
